# The Impact of Functional Stability Exercises on Alleviating Pelvic Girdle Pain in Pregnancy: A Review

**DOI:** 10.7759/cureus.48769

**Published:** 2023-11-13

**Authors:** Saurabh N Puri, Tejaswini Fating, Prasad P Dhage

**Affiliations:** 1 Department of Community Health Physiotherapy, Ravi Nair Physiotherapy College, Datta Meghe Institute of Higher Education and Research (Deemed to Be University), Wardha, IND

**Keywords:** functional stability exercises, pubis symphysis dysfunction, pregnancy, low back pain, pelvic girdle pain

## Abstract

Pelvic girdle pain (PGP) during pregnancy is a major source of stress for mothers. This review summarizes studies on the effectiveness of functional stability exercises (FSEs) in preventing PGP during pregnancy. FSE is a rising area of study in maternal health, focusing on core muscle groups and addressing the biomechanical changes during pregnancy. Although data shows that FSE may relieve PGP and improve the quality of life in pregnant women, the research landscape is defined by limitations and differences in intervention parameters among studies, resulting in contradictory conclusions. As a result, the efficacy of FSE in pregnant women with PGP remains inconclusive. This review can help comprise the existing research on FSE alleviating PGP in pregnancy to provide full knowledge on the topic, analyze long-term effects, and develop practice guidelines. While FSE shows promise, treating the multidimensional nature of PGP in pregnancy requires a comprehensive approach to therapy that incorporates several therapeutic modalities.

## Introduction and background

A musculoskeletal disorder known as pelvic girdle pain (PGP) affects women who are pregnant or recently gave birth. Symphysis pubis dysfunction (SPD) is a form of PGP and is used interchangeably in patients with SPD. Academics and healthcare practitioners have used different terminology to differentiate PGP from lower back pain related to pregnancy. According to European diagnostic criteria, the pain felt between the posterior iliac crest and the gluteal fold is referred to as PGP. It usually affects the sacroiliac joints (SIJs) but may also spread to the groin, perineum, or back of the thigh. PGP lacks a clear nerve distribution pattern, in contrast to traditional nerve root pain [1]. PGP is a condition that is frequently overlooked in pregnant and postpartum women. According to statistics, it occurs at 63% during the 30th gestational week, 31% three months postpartum, and 30% one year after delivery [2]. Symptoms often emerge in the second part of a pregnancy, peaking between the sixth and ninth months [3]. PGP can range in severity from minor to severe, creating disturbances in activities of daily living. It reduces endurance in sitting, walking, standing, and duties like lifting, changing postures, and getting up from seats, affecting women's quality of life severely [4].

PGP has been linked to psychological and social effects on women, limiting physical activity and social interaction resulting in feelings of despair and loneliness [5]. This pain can have a detrimental impact on afflicted women's quality of life, and there is some evidence of economic loss, as a result of missed work [6,7]. Patients with PGP and back pain exhibit unique, recognizable, and regular motion patterns [8]. PGP has been associated with several risk factors, including general hypermobility, prior pelvic trauma, age, a large number of previous births, and a high body mass index during pregnancy and before pregnancy [9-11]. PGP and/or pregnancy-related low back pain (PLBP) have been linked to several characteristics, including intense employment, past low back pain (LBP) experiences, and a history of PGP or PLBP, all of which are strong predictors of PGP/PLBP. Disorders can lead to aberrant cellular processes, mechanical stress, or inflammation in a specific region, all of which can result in localized tissue damage. The injury has the potential to impair normal tissue function and cause symptoms specific to the injured area to arise [3]. Contraceptive pill use, the time since the previous pregnancy, height, body weight, smoking, and age are all non-risk factors.

Functional stability exercises (FSEs) emphasize methodical and repetitive routines while focusing on strengthening particular body regions. They are organized physical activities. Physiotherapists recommend managing PGP with dynamic control exercises that activate both local and global pelvic girdle muscles [1]. Local stabilizing muscles, such as the transverse orientated abdominal muscles, lumbar multifidus, and pelvic floor muscles (levator ani, bulbospongiosus, ischiococcygeus, puborectalis, coccygeus, and pubococcygeus), are involved in lumbar load transmission. PGP is connected with issues with lumbopelvic load transmission. Targeting specific muscle areas, especially the pelvic and core muscles, stabilizing exercises increase strength. By using these muscles and conditioning them through regulated, repetitive motions, the workouts eventually build up the muscles' strength and endurance. Individuals can better manage everyday activities and lessen the pressure on their pelvis by strengthening the stability and support these muscles give, which will lessen the symptoms of PGP [12]. There is also the use of several non-invasive therapeutic techniques in the management of PGP. Manual therapy and other treatments such as acupuncture and pelvic belts are examples of passive treatment alternatives [13,14]. More active therapy options include specialized workouts with techniques to both increase or reduce muscle activation [15,16]. The purpose of the systematic review was to look into the efficacy of physiotherapy techniques in treating postpartum LBP and PGP.

## Review

Data sources and search engine

The study included original studies, systematic reviews, meta-analyses, and randomized controlled trials, among other sorts of academic materials. These articles were found using a combination of keywords and Medical Subject Headings phrases. Keywords were also used to narrow down the list of articles. Keywords like pelvic girdle discomfort, LBP, pregnancy-related PGP, PLBP, pregnancy-related lumbopelvic pain, and symphysis pubis dysfunction were used to select publications. Web search engines PubMed, Scopus, Web of Science, and Google Scholar were used to find the articles.

Methodology

**Figure 1:**
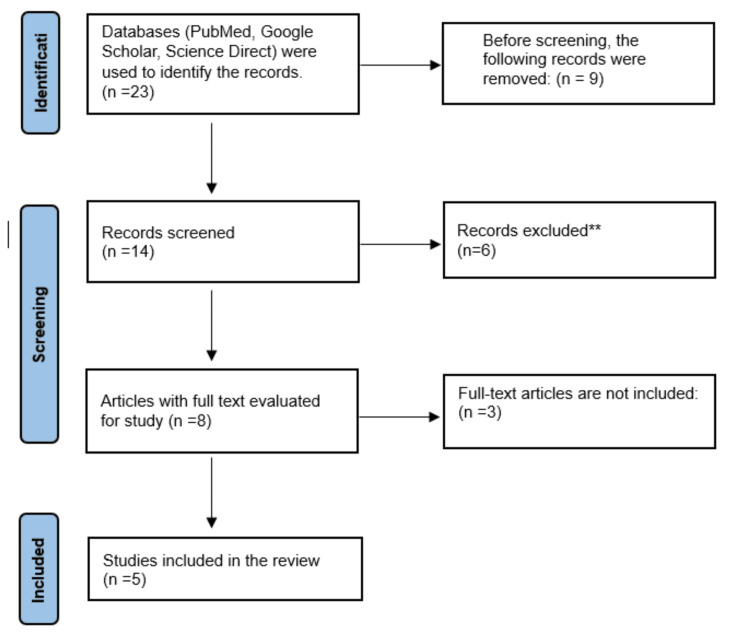
An overview of publications that are selected

A total of 23 records of systematic literature were examined for eligibility as full-text publications. After removing nine unrelated studies and nine duplicates, this review includes a total of five papers, which are listed below. One of the key reasons for exclusion was the inability to access the full version of articles. As a result, additional literature on the subject is required. There were numerous sorts of studies, including experimental studies, randomized controlled trials, systematic reviews, and literature reviews. Figure 1 depicts a summary of the articles chosen in compliance with Preferred Reporting Items for Systematic Reviews and Meta-Analysis guidelines.

Pelvic girdle pain

It can be challenging for patients and therapists to pinpoint the precise reason for pelvic discomfort during pregnancy since it can result from a multitude of factors. There is no widely accepted cause or cure for persistent PGP. This is because PGP is merely a symptom of another issue which includes sprain, strain, subluxation, abnormal spinal alignment, muscle asymmetry, weakness, tightness, etc [3]. PGP is a musculoskeletal condition that affects the gluteal fold, posterior iliac crest, and anterior and posterior bony pelvis [17]. Between the conclusion of the first trimester and the first month following delivery, PGP might manifest at any time [18]. Soft tissue or bone infections, lower back pain issues, pregnancy, joint hypermobility, and connective tissue abnormalities are all possible causes of pelvic discomfort [19]. Joint problems might impede everyday chores and life quality. PGP includes a wide range of symptoms such as lumbopelvic pain, symphysis pubis dysfunction, and pregnancy-related discomfort [20]. About 8-20% of women have agonizing pain two to three years after giving birth due to one of these disorders [21].

When assessing PGP, healthcare providers often look at a cluster of symptoms that contribute to the diagnosis. These symptoms encompass a wide range of discomforts and sensations that the person is experiencing. A wide sense of discomfort and pain over the pelvic girdle area, appearing as aching, soreness, or tenderness, is one of them. Furthermore, recognizing certain pain patterns, such as posterior pain localized in the region of the SIJs, which are the connections between the sacrum and the ilium bones of the pelvis, is also required for PGP diagnosis. Another differentiating feature is pain in the pubic symphysis, which connects the two pubic bones at the front of the pelvis. Furthermore, people with PGP may have radiating discomfort down the inner thigh, which typically follows the path of nerves and muscles that connect to the pelvic area. A thorough evaluation is necessary to correctly diagnose and treat PGP because of its complex clinical picture and many symptoms [19].

Functional stability exercises

FSEs are a sort of organized physical activity with the main objective of strengthening particular body parts. They are distinguished by their systematic and repeated nature. The goals of this conditioning process include maintaining and enhancing physical fitness levels as well as fostering overall health and well-being. Exercise is also a vital tool for those who want to recover after illnesses, operations, or accidents. Its numerous advantages include improving cardiovascular health, increasing physical strength and endurance, increasing flexibility, and improving mental well-being. In essence, exercise is crucial in promoting a holistic approach to health, embracing both the physical and mental components, making it an essential component of a balanced and healthy lifestyle [22]. The first-line treatment for chronic lower back pain has been proposed to be supervised exercise therapy throughout the past 10 years. However, during the past 10 years, stabilizing exercises and motor control drills have been touted as the best workouts for LBP and PGP worldwide [23]. Exercises for stabilization that depend on the principles of motor learning target certain core muscles that control the motion of the segments of the spine. These exercises use motor learning to improve muscle control and coordination, which improves the stability and function of the spine. Their mission is to assist patients in regaining control and coordination of the spine and pelvis [24]. For many years, academics, fitness centers, patients, and physicians have focused on core stabilizing activities. Although stabilizing activities have grown in popularity, the data is mixed. Some recent studies find that stabilization exercises are more helpful than general exercises, whereas others disagree [25]. Pregnancy-related PGP is treated through particular FSEs that target the physiological changes that occur during pregnancy. By strengthening supporting muscles, improving pelvic stability, and improving posture, these exercises lessen discomfort by better supporting the pelvic area and easing pressure on impacted tissues. They use moderate, regulated motions to develop core muscles (abdomen, back, pelvic floor) while emphasizing appropriate posture. These exercises are customized based on the needs of the person and may include modified squats, mild abdominal bracing, and pelvic tilts. Consultation with a healthcare physician or pregnant exercise instructor is vital for pregnancy safety and effectiveness.

Treatment

Patient education, especially targeted education and training programs, has been shown to reduce work absenteeism among women suffering from back pain. Such programs, however, do not provide the same advantages for women suffering from PGP [26]. Women who participate in back care courses learn about anatomy, ergonomics, maintaining good posture, managing pain, and employing relaxation techniques. The prevention of tiredness, avoiding twisting when lifting, and avoiding taxing postures are advised for pregnant women who are experiencing back discomfort. They should prioritize maintaining a straight posture and getting regular breaks. In addition, women with PGP should avoid bouncing, jarring movements, uneven weight distribution on the legs (such as when getting dressed), hip abduction, and tasks that place a lot of pressure on the joints. It is suggested that the knees be flexed and pressed together when turning in bed [27,28]. A nest-shaped cushion, for example, is useful in lowering discomfort and sleep problems during the later stages of pregnancy. This cushion gives abdominal support to a lady who is lying on her side. Other options include employing a lumbar roll behind the lower back (when lying down with slightly raised feet), abdominal-lumbar supports, and sacroiliac belts [29]. Instead of being placed at the symphysis pubis, pelvic belts work best when worn somewhat below the anterior superior iliac spines [30]. Pelvic tilts help to improve pelvic alignment and stability and have a special function in managing PGP during pregnancy. The purpose of gentle abdominal bracing is to strengthen the core muscles. Kegel exercises, particularly during pregnancy, are indicated to promote pelvic floor stability. Hip circles increase hip joint mobility and can help with PGP pain. Leg lifts help to develop the hip muscles while also improving stability. Modified squats emphasize lower-body strength and help to enhance pelvic stability. Exercises using a stability ball are excellent for improving core stability and posture. Pelvic bridging exercises work the glutes and lower back muscles, which help to maintain pelvic stability [26].

Discussion

The purpose of the current study was to look at how stabilizing exercises affected PGP associated with pregnancy. Although there is some evidence that these activities can improve quality of life and lessen discomfort, there is not enough information available for women who are pregnant or just gave birth, so it is difficult to make firm judgments. There are several reasons for this intricacy. First, discrepancies in the results might have been caused by differences in the intervention parameters used in various research. To give a more thorough knowledge of the effects of stabilizing activities in this particular demographic, additional study is required [31]. In maternal health, FSEs for pregnancy-related PGP are gaining popularity. PGP, which is prevalent throughout pregnancy, can cause modest discomfort to severe pain, emphasizing the importance of appropriate care. These workouts target core muscles and address biomechanical changes that occur during pregnancy, perhaps lowering PGP severity and improving pelvic stability. Assessment by a healthcare expert is critical for safety, especially for novices. It is useful to incorporate these exercises into a complete care plan, although additional study is needed for standardized procedures and long-term benefits. Different research on the effect of FSEs on PGP is included in Table 1 below.

**Table 1:** Several studies have been conducted to determine the effectiveness of functional stability exercises for pelvic girdle pain

Author and year	Design	Sample size	Intervention and outcome measure	Conclusion
Stuge et al. [15]	A randomized controlled trial	81 women with PGP were assigned	The first group received targeted physical therapy that included stabilizing exercises, whereas the second group received tailored physical therapy that did not include stabilizing activities. The major outcomes investigated were pain, functional status, and quality of life.	For women with pelvic girdle discomfort after pregnancy, customized treatment techniques that incorporate specialized stabilizing exercises have proven to be more beneficial than physical therapy alone.
Elden et al. [31]	Randomized single-blind controlled trial	386 pregnant women with PGP	A pelvic belt, a personalized home training plan to improve abdominal and gluteal muscle strength, and acupuncture therapy were given to patients.	When combined with the standard therapeutic method for pelvic girdle discomfort during pregnancy, acupuncture, and stabilizing exercises are useful.
Gutke et al. [32]	A randomized controlled trial	There were 88 women with pelvic girdle discomfort.	Training included particular stabilizing exercises that targeted the transversely orientated abdominal, lumbar multifidus, and pelvic floor muscles.	For postpartum PGP, home-based stabilizing activities did not outperform the natural course. A year after delivery, most women, whether treated or not, still reported back discomfort.
Kordi et al. [33]	A randomized controlled trial	105 pregnant women experiencing PGP	Pregnant women experiencing pelvic girdle discomfort were randomized to one of three groups at random: control (n=35, general information), exercise (n=31, home exercises plus info), or belt (n=31, belt + info).	Pregnant women experiencing pelvic girdle discomfort were given general information and told to do specific pelvic stabilization exercises at home while wearing a non-rigid lumbopelvic belt.
Ozdemir et al. [34]	Randomized controlled trial	96 pregnant women	Four-week training comprised both mattress-based and walking exercises for vast muscle groups from the neck to the spine.	A four-week exercise program that included individualized health advice to address low back and pelvic discomfort enhanced pregnant women's functional status.

## Conclusions

In conclusion, the study on functional stability exercises (FSE) for PGP during pregnancy indicates a complicated subject in maternal healthcare. While there is evidence that FSE can help lessen PGP discomfort and improve pregnant women's quality of life, study limitations remain. The evidence is fairly restricted, particularly for pregnant and postpartum women, resulting in contradictory findings and difficulties in drawing broad generalizations regarding the efficacy of FSE. Given the variable intensity of PGP during pregnancy, it is critical to recognize FSE's potential in maternal health. These workouts focus on core muscles and pregnancy-related biomechanical changes, which may help to reduce PGP severity and improve pelvic stability. However, it is critical to emphasize the need for individualized assessments by healthcare experts, particularly for newcomers, with a focus on pregnancy safety. Further study is needed to develop standardized methods, examine long-term effects, and give a full knowledge of FSE's involvement in controlling PGP throughout pregnancy. While FSE shows potential, treating the multidimensional nature of PGP in pregnancy requires a holistic treatment strategy that incorporates several interventions.
